# Apples as Medicine

**Published:** 1890-07

**Authors:** 


### APPLES AS MEDICINE.

We take the following from a late *Phrenological Journal*, and we are able to endorse all that is said in praise of the commonest and best of our northern fruits. The truth is, we are growing more and more in favor of a vegetable diet for all brain workers and persons of sedentary habits, and we are by no means sure that such a course of living is not the best for all classes.

We do not know why a common fruit like apples, or indeed any fruit, should be talked of as medicine, any more than bread and butter, unless it is the fact that people have not been in the habit of eating enough fruit to meet the want of the digestive function for natural or organic acids. That apples contain elements that also enter into leading drugs is true.

Chemically, the apple is composed of vegetable fiber, albumen, sugar, gum, chlorophyll, malic acid, gallic acid, lime, and much water. Furthermore, the German analysts say that the apple contains a larger percentage of phosphorus than any other fruit or vegetable. This phosphorus is admirably adapted for renewing the essential nervous matter, lethicin, of the brain and spinal cord. It is, perhaps, for the same reason, rudely understood, that old Scandinavian traditions represent the apple as the food of the gods, who, when they felt themselves to be growing feeble and infirm, resorted to this fruit for renewing their powers of mind and body.

The acids of the apple are of use for men of sedentary habits, whose lives are sluggish in action ; these acids serving to eliminate from the body, noxious matters which, if retained, would make the brain heavy and dull, or bring about jaundice or skin eruptions and other allied troubles. Some such an experience must have led to our custom of making apple sauce with roast pork, rich goose, and like dishes. The malic acid of ripe apples, either raw or cooked, will neutralize any excess of chalky matter generated by eating too much meat. It is also the fact that such fresh fruit as the apple, the pear, and the plum, when taken ripe and without sugar, diminish the acidity in the stomach rather than provoke it. Their vegetable salts and juices are converted into alkaline carbonates, which tend to counteract acidity. A good ripe, raw apple is one of the easiest of vegetable substances for the stomach to deal with, the whole process of its digestion being completed in eighty-five minutes.

Gerard found that the "pulp of roasted apples mixed in a wine quart of faire water, and labored together until it comes to be as apple and ale—which we call lambswool—never faileth in certain diseases of the raines, which myself hath often proved, and gained thereby both crownes and credit." "The paring of an apple, cut somewhat thick, and the inside thereby is laid to hot, burning, or running eyes at night, when the party goes to bed; and is tied, or bound to the same, doth help the trouble very speedily, and contrary to expectation—an excellent secret." A poultice made of rotten apples is of very common use in Lincolnshire for the cure of weak or rheumatic eyes. Likewise, in the Hotel des Invalides, at Paris, an apple poultice is commonly used for inflamed eyes, the apple being roasted and its pulp applied over the eyes without an intervening substance. A modern maxim teaches that—"To eat an apple going to bed, the doctor then will beg his bread."

